# Rational Development of A Polycistronic Plasmid with A CpG-Free Bacterial Backbone as A Potential Tool for Direct Reprogramming

**DOI:** 10.22074/cellj.2016.4723

**Published:** 2016-09-26

**Authors:** Kianoush Dormiani, Hamid Mir Mohammad Sadeghi, Hojjat Sadeghi-Aliabadi, Mahboobeh Forouzanfar, Hossein Baharvand, Kamran Ghaedi, Mohammad Hossein Nasr-Esfahani

**Affiliations:** 1 Department of Pharmaceutical Biotechnology and Isfahan Pharmaceutical Sciences Research Center, School of Pharmacy and Pharmaceutical Sciences, Isfahan University of Medical Sciences, Isfahan, Iran; 2 Department of Molecular Biotechnology, Cell Science Research Center, Royan Institute for Biotechnology, ACECR, Isfahan, Iran; 3 Department of Stem Cells and Developmental Biology, Cell Science Research Center, Royan Institute for Stem Cell Biology and Technology, ACECR, Tehran, Iran; 4 Department of Developmental Biology, University of Science and Culture, ACECR, Tehran, Iran; 5 Department of Biology, School of Sciences, University of Isfahan, Isfahan, Iran

**Keywords:** 2A Peptide, CpG Dinucleotide, Extrachromosomal Plasmid, Polycistronic, Reprogramming

## Abstract

**Objective:**

Induced pluripotent stem cells are generated from somatic cells by direct reprogramming. These reprogrammed pluripotent cells have different applications in biomedical fields such as regenerative medicine. Although viral vectors are widely used for efficient reprogramming, they have limited applications in the clinic due to the risk for immunogenicity and insertional mutagenesis. Accordingly, we designed and developed a small, non-integrating plasmid named pLENSO/Zeo as a 2A-mediated polycistronic expression vector.

**Materials and Methods:**

In this experimental study, we developed a single plasmid which includes a single expression cassette containing open reading frames of human *LIN28, NANOG, SOX2* and *OCT4* along with an *EGFP* reporter gene. Each reprogramming factor is separated by an intervening sequence that encodes a 2A self-processing peptide. The reprogramming cassette is located downstream of a CMV promoter. The vector is easily propagated in the *E. coli* GT115 strain through a CpG-depleted vector backbone. We evaluated the stability of the constructed vector bioinformatically, and its ability to stoichiometric expression of the reprogramming factors using quantitative molecular methods analysis after transient transfection into HEK293 cells.

**Results:**

In the present study, we developed a nonviral episomal vector named pLENSO/ Zeo. Our results demonstrated the general structural stability of the plasmid DNA. This relatively small vector showed concomitant, high-level expression of the four reprogramming factors with similar titers, which are considered as the critical parameters for efficient and consistent reprogramming.

**Conclusion:**

According to our experimental results, this stable extrachromosomal plasmid expresses reliable amounts of four reprogramming factors simultaneously. Consequently, these promising results encouraged us to evaluate the capability of pLENSO/Zeo as a simple and feasible tool for generation of induced pluripotent stem cells from primary cells in the future.

## Introduction

Induced pluripotent stem cells (iPSCs) are generated from various human primary cells by ectopic expression of a number of exogenous transcription factors (1,2). The resultant iPSCs can be used for a variety of purposes and have great potential for use in regenerative medicine (3). These cells may act as a stable source of lineagespecific somatic cells due to their unique ability to self-renew and differentiate into a diverse range of somatic cell types (4,5). Precise selection of reprogramming vehicles is the key point to improve efficiency and safety in the generation of iPSCs, which requires sufficient knowledge of vectors and techniques for transgene delivery. In many research studies, integrating vectors such as retroand lentiviral vectors are still used for reprogramming because of providing sustained expression of transgenes that are silenced at the end of reprogramming process (6,7). 

It has been reported that viral vectors increase the risk of insertional mutagenesis and tumor formation due to their multiple, random integration into the genome of transduced cells. They can also lead to initiation of immune responses (2,8,9). Besides, some studies have shown that the probable residual expression or reactivation of exogenous reprogramming factors (RFs) during cell culture or differentiation may lead to destabilization of induced pluripotency that results in partially reprogrammed cells and interfere with differentiation capacity (10,12). To address these safety issues, alternative approaches such as non-integrating vectors, excisable vectors and DNA-free delivery of mRNAs or peptides of RFs have been developed for successful generation of virus-free iPSCs with its own advantages and disadvantages (13,16). 

Among different tools for reprogramming, a simple approach relies on use of the mixture of plasmids that are readily accessible by any laboratory (17). An attractive plasmid-based vector for iPSCs induction involves the implementation of a single non-integrating polycistronic vector that simultaneously expresses defined transcription factors using a 2A-mediated separation technique. The efficiency of the 2A peptide cleavage is sufficient to be used for co-expression of up to five heterogeneous genes (18,19). Appealing characteristics of this single-vector approach include minimal risk of genomic integration and a reduced vector size that provides better transfection efficiency. Also, balanced expression of discrete RFs by this type of vector improves the efficiency of reprogramming and reduces variability amongst generated iPSCs (19). There are a number of reports regarding the successful application of these polycistronic vectors for cellular reprogramming (14,20,23). Although this type of plasmid shows a number of advantages compared to conventional plasmids, some considerations should be taken into account in order to maximize reprogramming efficiency as described below. According to previous studies, the plasmid DNA (pDNA) size is a potent modulator in the efficiency of gene transfer and expression (24). Plasmid size clearly affects the efficiency of nuclear uptake; the smaller the plasmid size, the greater the transfection efficiency of the target cells (25). Larger plasmids are responsible for not only silencing transgenes, but also higher genomic integration events (26,27). 

On the other hand, pDNA topology is critical for gene delivery and transfection efficiency in mammalian cells (28). It is generally believed that the supercoiled, covalently closed circular form of pDNA is biologically active and has the highest transfection efficiency and transgene expression level compared to other isoforms, independent of cell type (29,30). Another consideration during development of a new plasmid is its stability and structural integrity. Certain sequences such as repeated DNA motifs and AT-rich fragments (cleavage hot-spots) contribute negatively to structural stability and transfection efficiency. The abundance of theses hot spots reduces the supercoiled isoform content due to the generation of nicks in theses sequences by cellular nucleases (31). Therefore, after plasmid preparation, it is reasonable to determine the content of the DNA supercoiled isoform as a parameter for assessing structural integrity. 

In light of biosafety issues and transgene expression profiles, one of the seminal aspects that require attention is the distribution of CpG motifs through the vector. In the mammalian cell nucleus, CpG dinucleotides are of low frequency throughout the genome with the exception of short fragments known as CpG islands. These islands contain GpC-rich stretches of DNA frequently mapped in gene regulatory elements, particularly promoters (32,33). Chen et al. (26) have demonstrated that the covalent linkage of the bacterial backbone (BB) sequences to the expression cassette is the main cause for transcriptional silencing. Sequences in the BB can act as centers for heterochromatin formation which subsequently spread to adjacent sequences and lead to silencing of the neighboring expression cassettes (34,35). It seems that unmethylated bacterial CpGs are responsible for epigenetic silencing events (36). Accordingly, new strategies have been employed to remove the BB or perform some modifications that result in reduction or elimination of the CpG motifs from the vector DNA (37,38). 

In the present study, we have developed a polycistronic vector, pLENSO/Zeo. The vector structural elements comprise: i. A multicistronic expression cassette and ii. A short bacterial propagation unit. The expression (reprogramming) cassette composed of four open reading frames (ORFs) human *LIN28, NANOG, SOX2* and *OCT4* in addition to the enhanced green fluorescent protein (EGFP) reporter gene that allows direct visualization of vector expression. These transcription factors (Thomson factors) (2) are fused to each other with intervening sequences that encode 2A self-cleaving peptides. A single human cytomegalovirus (CMV) promoter as a strong, constitutive promoter is located upstream of the reprogramming cassette. The CpG-free BB enables the vector to amplify in *E. coli* GT115 due to a modified R6K gamma-origin core replicon (R6Kγ), an EM2K promoter and a Zeocin resistance gene (*Zeo^r^*). We have evaluated the expression level of the reprogramming cassette by transfecting human embryonic kidney cells (HEK293) cells with the pLENSO/Zeo. Our results showed high transfection efficiency of the vector and confirmed concordant high-level expression of the four discrete RFs. 

## Materials and Methods

In this experimental study, we first amplified the ORFs of human *OCT4, SOX2, NANOG* and *LIN28* by reverse transcription-polymerase chain reaction (RT-PCR) using total RNA extracted from Royan H6 human embryonic stem cells (hESCs) (39) and appropriate primers (Table 1). All restriction enzymes were obtained from Thermo Scientific, USA. The primers were designed to introduce T2A sequences with appropriate restriction sites at the 3´ end of *LIN28, NANOG* and *SOX2* ORFs. The forward primer of *LIN28* ORF contained a Kozak consensus sequence that enclosed the ATG codon at the beginning of *LIN28* ORF for maximal translation. The downstream primer of *OCT4* carried two stop codons to ensure correct termination and limit read through translation. EGFP coding sequence along with T2A and SV40 polyadenylation (SV40PA) signal sequences were separately amplified from plasmid pEGFP-C1 (Clontech Laboratories, USA).

All ORFs were separately inserted into the pTZ57RT (Thermo Scientific, USA) through T/A cloning. The pTZ/OCT4 was double digested with SalI and SmaI. An isolated OCT4 fragment was subcloned into pTZ/SOX2 instead of the XhoI-SmaI fragment downstream of the *SOX2* ORF to produce the pTZ/SOX2/OCT4 plasmid. Next, *NANOG* ORF was digested using EcoRI and BglII, and subcloned instead of EcoRI-BamHI fragment located upstream of SOX2 in pTZ/SOX2/OCT4, which resulted in the creation of pTZ/NANOG/SOX2/OCT4. The pTZ/LIN28 was also digested with XhoI and EcoRI, and the XhoI-LIN28-EcoRI fragment was then subcloned into compatible sites (SalI and EcoRI) upstream of the EGFP in pTZ/EGFP. We named the resultant vector pTZ/LIN28/EGFP.

By digesting pTZ/NANOG/SOX2/OCT4 with AgeI and SmaI, NANOG/SOX2/OCT4 fragment was isolated and inserted at the same place in pTZ/LIN28/EGFP downstream of EGFP. This reaction produced pTZ/LIN28/EGFP/NANOG/SOX2/OCT4 which was digested by NheI and SmaI to isolate LIN28/EGFP/NANOG/SOX2/OCT4. This fragment, hereafter termed LENSO, was subcloned into the digested pEGFP-C1 downstream of the human CMV promoter that generated a new vector named pLENSO-C1. Subsequently, pTZ/SV40PA was digested by SmaI and XbaI. A gel extracted SV40PA signal fragment was inserted into pLENSO-C1 downstream of the OCT4 sequence. The resultant recombinant vector was named pLENSO-PA. To remove the CpG motifs in BB, three fragments of pCpG-free basic plasmid that contained an EM2K prokaryotic promoter, *Zeo^r^* and R6Kγ ori (OriZeo) were amplified from a pCpG-free basic plasmid (InvivoGen, USA) using NdeIFori as the forward primer and NdeIRzeo as the reverse primer (Table 1). The 700 bp-amplified product was T/A cloned which created pTZ/ OriZeo, and then isolated following AseI digestion. The AseI-OriZeo-AseI fragment was inserted into pLENSO-PA in place of NdeI-BBNdeI. The final recombinant vector, pLENSO/ Zeo, was transformed into the competent *E. coli* GT115 (InvivoGen, USA). The transformation mixture was spread on Fast-Media Zeo agar (InvivoGen, USA) as a Zeocin selection medium. The colony which contained pLENSO/Zeo was identified by direct colony PCR and plasmid purified using the EndoFree Plasmid Maxi Kit (Qiagen, Germany) and then stored at 2-4˚C. Final purified pDNA was digested with NotI and HindIII separately and double digested with HindIII and NotI. Restriction digestion patterns were used for confirmation of the plasmid size. It is noteworthy that each amplified fragment was verified by sequencing to avoid any mutation.

**Table 1:** List of primers used for construction of the polycistronic vector


Primer name	Sequence

EcoRI-NheI F-Lin28	GAATTCCTCGAGATGGGCTCCGTGTCCAAC
XhoI 2A R-Lin28	CATATGGGTGGCGGCCGCAGGGCCGGGATTCTCCTCCACGTCACCGCATGTTAGAAGACTTCCTCTGCCCTCCACCGGTATTCTGTGCCTCCGGGAG
EcoRI-AgeI F-Nanog	GAATTCACCGGTATGAGTGTGGATCCAGCTTG
BglII 2A R-Nanog	AGATCTAGGCGGCCGCAGGGCCGGGATTCTCCTCCACGTCACCGCATGTTAGAAGACTTCCTCTGCCCTCACCGGTCACGTCTTCAGGTTGCATG
BamHI F-Sox2	GGATCCATGTACAACATGATGGAGACG
XhoI 2A R-Sox2	CTCGAGAGGGCCGGGATTCTCCTCCACGTCACCGCATGTTAGAAGACTTCCTCTGCCCTCCACCGGTCATGTGTGAGAGGGGCAG
SalI F-Oct4	GTCGACATGGCGGGACACCTGGCTTC
SmaI R-Oct4	CCCGGGCTATCAGTTTGAATGCATGGGAGAGC
SalI F-EGFP	GGTACCATGGTGAGCAAGGGCGAG
AgeI 2A R-EGFP	ACCGGTAGGGCCGGGATTCTCCTCCACGTCACCGCATGTTAGAAGACTTCCTCTGCCCTCCACCGGTCTTGTACAGCTCGTCCATGC
SmaI F-SV40PA	CCCGGGCATAATCAGCCATACCAC
AseI R-SV40PA	ATTAATTAAGATACATTGATGAGTTTGG
NdeI F-Ori	CATATGAATCAGCAGTTCAACCTG
NdeI R-Zeo	CATATGTTGACAATTAAACATTGGCATAG


Specific restriction site(s) in each primer is underlined.

### Bioinformatic analysis of the reprogramming vector

One of the major problems encountered during the construction and propagation of a vector is protection of its structural integrity. Potentially unfavorable motifs lead to destabilization of the pDNA. To distinguish whether this concern was applicable to our reprogramming vector prior to *in vitro* analysis, we have estimated the stress-induced duplex destabilization (SIDD) energy through the following web-based tool WebSIDD (http://benham.genomecenter.ucdavis.edu/sibz/). The user enters the sequence of pDNA and the program estimates the transition probability and destabilization energy for each base pair in the target sequence (40). G(x) is the denaturation energy (kcal/mol) needed to force the base pair at position x to open in supercoiled DNA. Stable positions in the vector have high values of G(x) close to 10, whereas unstable positions have low values and are prone to degradation by cellular nuclease attack (41). Additionally, the position of each CpG dinucleotide in the plasmid has been identified by the EMBOSS fuzznuc tool (http://emboss.bioinformatics.nl/cgi-bin/emboss/fuzznuc). To plot the respective graph, we divided the sequence of our recombinant vector into 26 fragments of 250 bp each and estimated the number of CpG motifs in each fragment. 

### Topological studies of the reprogramming vector

We isolated topological isoforms of pLENSO/ Zeo by loading 300 ng of undigested plasmid beside 150 ng of NotI digested plasmid onto a 1.0% agarose slab gel. Gel electrophoresis was performed in TAE buffer (20 mM Tris, 10 mM acetic acid and 0.5 mM EDTA, pH=8.0) at a constant voltage of 60 V at room temperature using the gel electrophoretic unit, Wide Mini-Sub Cell GT Cell (Bio-Rad Laboratories, USA). The gel was stained with ethidium bromide and visualized by UV light. The gel image was captured using a transilluminator, UVITEC ESSENTIAL V2 (UVItec, UK) that contained a charge-coupled device (CCD) grade camera. The intensity of the resultant DNA bands was quantified by optical densitometry using NIH ImageJ software version 1.48 (http://rsb.info.nih.gov/ij/). 

### Cell culture, transfection and enhanced green fluorescent protein expression

HEK293 cells (CRL-1573, ATCC, USA) were used as a model for transfection with pLENSO/Zeo in order to functionally test the vector for accurate expression of transgenes. The cells were cultured in medium that contained DMEM, high glucose supplemented with 4 mM L-glutamine, 10% fetal bovine serum, 100 U/ mL penicillin and 100 µg/mL streptomycin (all from Gibco, USA). One day prior to transfection, approximately 5×10^5^ cells were seeded in each gelatin-coated 60 mm plate. On transfection day, when the cell density reached 70-80%, the medium was refreshed without antibiotics 3 hours before cell transfection. For transfection of each plate, we used 7 µg of pLENSO/ Zeo DNA diluted in Opti-MEM I Reduced Serum Medium (Gibco, USA) and 21 µL of Lipofectamine LTX reagent (Invitrogen, USA) according to the manufacturer’s instructions. EGFP expression in transfected HEK293 cells was monitored at defined time points (1, 3, 5, 7, 10, 12 days post-transfection) by fluorescent microscopy and flow cytometry. 

### Flow cytometric analysis

The plasmid-induced GFP signal was assessed by fluorescence activated cell sorting (FACS) analysis as an indicator of stem cassette mediated-expression. In each experiment, the percentage of EGFP^+^ cells was measured by comparing approximately 1×10^6^ transfected HEK293 cells with reference to a baseline of non-transfected cells. The cells were trypsinized 24 hours post-transfection, washed with PBS and divided into two parts. One part was immediately used to measure EGFP expression in the fluorescence detector 1 (FL-1) with a 530/30 nm band pass filter by FACSCalibur (BD Biosciences, USA). For each sample 104 events were recorded and data analyzed by Cell Quest Pro software (Becton-Dickinson, USA). The remaining cells were cultured for an additional 10-12 days and used for FACS analysis to determine EGFP expression at the aforementioned time points after transfection.

### Detection of vector integration into the host genome

We assessed genomic integration of pLENSO/ Zeo by isolating genomic DNA from transfected HEK293 cells 12 days after transfection and from untransfected cells as the negative control. For this, approximately 1×10^6^ HEK293 cells were used to isolate each genomic DNA with the DNeasy Blood & Tissue Kit (Qiagen, Germany). Approximately 5 μg of transfected and untransfected genomes and the pLENSO/Zeo DNA were digested by NotI for a 3-hour incubation period at 37˚C. Digested samples were analysed by gel electrophoresis, after which the genomic samples were purified by a Gel Extraction Kit (Qiagen, Germany). Using 200 ng of each purified genomic samples and 80 ng pLENSO/ Zeo circular DNA as the templates, PCR was performed by ExTaq polymerase (Takara, Japan) in a 25-μL final volume. For PCR experiments, we used six pairs of primers to amplify all parts of the pLENSO/Zeo DNA. The list of primers and the expected product size from each are shown in Table 2. Amplification conditions were 95˚C for 2 minutes, 35 cycles of 95˚C for 30 seconds, 60˚C for 40 seconds and 72˚C for 90 seconds, followed by incubation at 72˚C for 10 minutes.

**Table 2:** List of primers used for genomic PCR analysis


Primer pair no.	Amplified segment	Primer name	Sequence (5´-3´)	Tm (°C)	size	
					Vector	Genome

1	Ori and LIN28	R: Ori	CATATGTTGACAATTAAACATTGGCATAG	66.2	1300	1300
		R: Lin28	CTGCCTCACCCTCCTTCAG	67.4		
2	LIN28 and EGFP	F: Lin28	GTTCGGCTTCCTGTCCATG	64.0	1200	-
		R: EGFP	GGTGCTCAGGTAGTGGTTGTC	68.6		
3	EGFP and NANOG	F: EGFP	CAAGCAGAAGAACGGCATCAAG	64.8	1350	-
		R: Nanog	TGGTGGTAGGAAGAGTAAAGG	64.3		
4	NANOG and SOX2	F: Nanog	CAGCTACAAACAGGTGAAGAC	62.5	1490	1500
		R: Sox2	CTCTGGTAGTGCTGGGACATG	68.0		
5	SOX2 and OCT4	F: Sox2	ACCTCTTCCTCCCACTCCAG	69.1	900	900
		R: Oct4	ATTGTTGTCAGCTTCCTCCA	62.0		
6	OCT4 and Ori	F: Oct4	TCTATTTGGGAAGGTATTCAGC	62.4	1700	-
		F: Ori	CATATGAATCAGCAGTTCAACCTG	63.7		


PCR; Polymerase chain reaction and Tm; Melting temperature.

### RNA extraction, reverse transcription and quantitative polymerase chain reaction

Two days post-transfection, the HEK293 cells were detached by TrypLE and collected by centrifugation at 1800 rpm for 5 minutes. The cells were lysed in 750 μL of TRI reagent (Sigma-Aldrich, USA) according to the manufacturer’s protocol. Total RNA was isolated, quantified and stored at -70˚C. For reverse transcription quantitative PCR (RT-qPCR), we synthesized the cDNAs using a RevertAid Premium First Strand cDNA Synthesis Kit (Thermo Scientific, USA) with random hexamer primers. All measurements were run in triplicate on a StepOnePlus RealTime PCR System (Applied Biosystems, USA) using the SYBR Green Master Mix (Takara, Japan). The cycling program was 95˚C for 30 seconds, followed by 40 cycles at 95˚C for 10 seconds, 60˚C for 30 seconds and 72˚C for 30 seconds. Specific primers were designed by Beacon Designer software (Version 7.2, USA) and used according to Table 3. Expressions of RFs were estimated by the comparative Ct method using glyceraldehyde 3-phosphate dehydrogenase (GAPDH) as the reference gene. In order to estimate the ectopic expression of each RF by the vector, transcription levels of endogenous LIN28, NANOG, SOX2 and OCT4 genes in untransfected HEK293 cells were assessed and subtracted from the total expression of corresponding RF in transfected cells. By acquiring the vector-mediated expression of four RFs, expression level of OCT4 peptide was considered to be 100% and the other RFs were compared to OCT4. 

### Western blot analysis

The most effective way to verify co-expression of four discrete transcription factors by a multicistronic plasmid is the transient transfection of HEK293 cells followed by quantitative Western blot analysis (19,42). Therefore, we collected 1×10^6^ cells 48 hours after transfection. Total protein of the cells was extracted using the TRI reagent (Sigma-Aldrich, USA). As the positive control, we used protein lysates from Royan H6 hESCs. Then, 35 μg of solubilized protein fraction of each sample was subjected to 12% SDS-PAGE and eletrophoretically transferred to a polyvinylidene difluoride membrane (PVDF, Bio-Rad Laboratories, USA). 

After blocking overnight with 10% (w/v) nonfat dried milk (Merck, Germany) in PBS buffer, the membranes were labeled with the following primary antibodies: rabbit anti-OCT4 (Santa Cruz, USA, sc5279, 1:1000), mouse anti-SOX2 (Santa Cruz, USA, sc-365823, 1:1000), goat anti-NANOG (Santa Cruz, USA, sc-30331, 1:1000), rabbit anti-LIN28 (Proteintech, USA, 11724-1-AP, lot 2, 1:500) and primary mouse anti-GAPDH (Millipore, USA, MAB374, 1:5000). The secondary antibodies were goat antimouse IgG (DakoCytomation, Denmark, P0447, 1:5000), donkey anti-goat IgG (Santa Cruz, USA, sc2020, 1:20000) and goat anti-rabbit IgG (Santa Cruz, USA, sc-2301, 1:16000) conjugated with horseradish peroxidase. The protein bands were visualized using an Amersham ECL Advance Western Blotting Detection Kit (GE Healthcare, Germany). In order to evaluate protein expression levels of the RFs, final images were acquired with a CCD camera and used for band analysis. The intensities of the acquired bands were quantified by ImageJ software (version 1.48, NIH) and normalized to the mean intensities of the GAPDH protein from each experiment. Finally, the mean of resultant intensity for each RF was compared to OCT4. 

**Table 3:** List of primers used for RT-qPCR


Target gene	Primer name	Sequence (5´-3´)	Expected size (bp)

*LIN28*	F: Lin28	GTTCGGCTTCCTGTCCAT	122
	R: Lin28	CTGCCTCACCCTCCTTCA	
*NANOG*	F: Nanog	CAGCTACAAACAGGTGAAGAC	146
	R: Nanog	TGGTGGTAGGAAGAGTAAAGG	
*SOX2*	F: Sox2	ACCTCTTCCTCCCACTCCAG	134
	R: Sox2	CTCTGGTAGTGCTGGGACATG	
*OCT4*	F: Oct4	TCTATTTGGGAAGGTATTCAGC	124
	R: Oct4	ATTGTTGTCAGCTTCCTCCA	


RT-qPCR; Reverse transcription-quantitative polymerase chain reaction.

## Results

### Design and construction of the reprogramming vector

**Fig.1:**
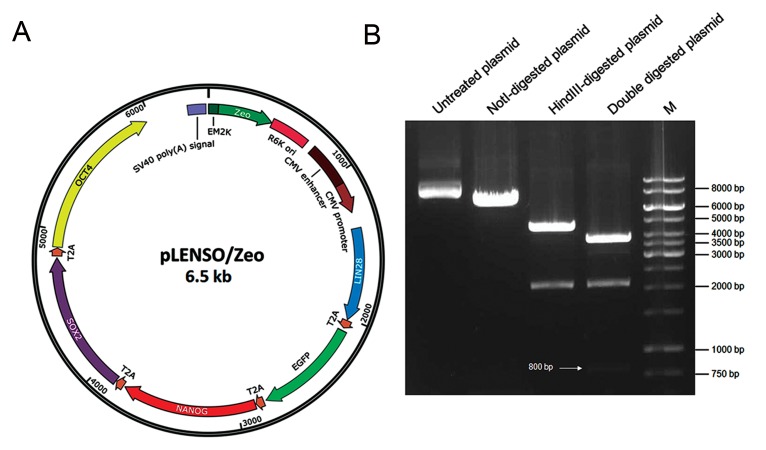
Design and construction of the reprogramming vector. A. Schematic representation of pLENSO/Zeo structure. The vector consists of a CpG-free vector backbone and a single expression cassette that has the capability to efficiently produce human *LIN28, NANOG, SOX2* and *OCT4* peptides in addition to the EGFP and B. Plasmid size was determined by restriction digestion map. The pDNA was digested with NotI (1 site), HindIII (2 sites) separately and also double digested with the two enzymes for 3 hours. Following electrophoresis, the agarose gel showed untreated plasmid, NotI-linearized plasmid with a single band of about 6500 bp and HindIII-digested plasmid fragments (2100 and 4400 bp). Besides, double digestion with NotI and HindIII resulted in three distinct bands with approximate sizes of 3600, 2100 and 800 bp indicated by a white arrow. The sum of these DNA fragments is 6500 bp consistent with the predicted size for constructed plasmid. M; Molecular size marker (1 kb) Plus DNA Ladder (Thermo Scientific, USA).

We developed a small reprogramming plasmid with RFs along with EGFP coding sequences located in a defined order in the stem cassette under the control of a single CMV promoter element. The vector consisted of a 700 bp CpG-depleted BB fragment that consisted of an EM2K prokaryotic promoter and *Zeo^r^* as well as R6K origin of replication (Fig .1A). We inserted the BB into the vector where the prokaryotic promoter was located distal to the eukaryotic promoter. The functionality of the BB in the pLENSO/Zeo vector was confirmed by its ability to propagate the plasmid in *E. coli* GT115 competent cells. After purification, linearization of the pDNA with NotI confirmed the size of constructed plasmid to be about 6500 bp. Moreover, the estimated plasmid size was also established by adding the size of resulted bands in the digestion with HindIII and double digestion of pLENSO/Zeo DNA (Fig .1B). Correct orientations of different cloned fragments in the vector and their accuracy were ascertained by PCR using appropriate primers and sequencing.

### Analysis of the reprogramming vector in terms of stability and CpG content

**Fig.2:**
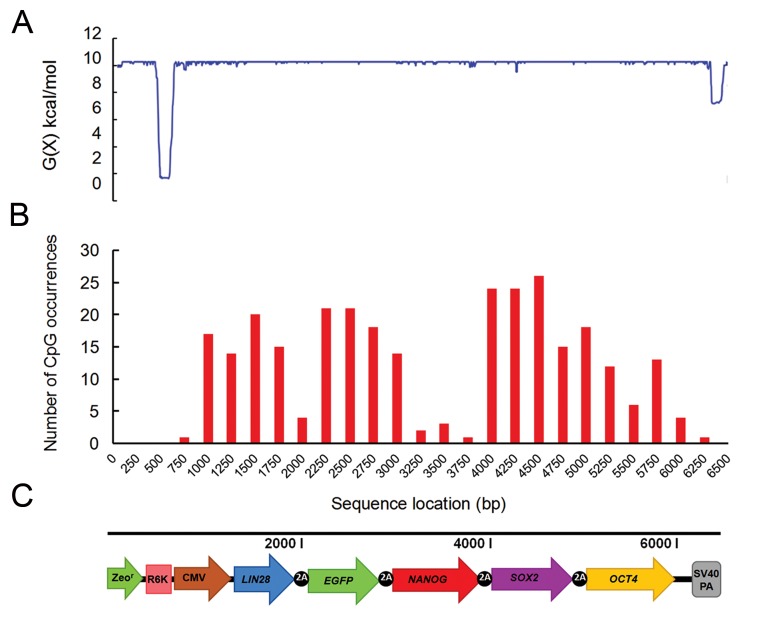
Bioinformatic analysis of pLENSO/Zeo vector. A. The destabilization energy profi le refers to the stress-induced duplex destabilization energy (SIDD). The energy cost [G(x), kcal/mol] leads to strand separation in conditions of negative DNA superhelicity. Values of G(x) close to 10.2 correspond to complete stability, B. The graph shows the distribution and relative abundance of CpG dinucleotides throughout the vector. A high abundance of these types of elements is particularly found within human SOX2 that harbors a total of 88 CpG motifs, while the bacterial backbone contains no CpGs, and C. Linear map of the pLENSO/Zeo plasmid.

In Figure 2, graph A, the locations and extent of G(x) values in pDNA molecule show that only two regions in the vector, R6Kγ ori and SV40PA signal, are AT-rich and demonstrate a propensity for duplex destabilization. These data suggest that the vector contains rare cleavage hotspots and hence most of the pDNA sequences remain virtually stable, similar to an unstressed molecule. Graph B shows the distribution and relative abundance of CpG dinucleotides in different regions of pLENSO/Zeo. As we expected, BB and SV40PA signals were CpG-free, while the reprogramming cassette totally contained 230 intragenic CpG dinucleotides. The CpG frequency within ORFs was as follows: LIN28=33, EGFP=60, NANOG=7, SOX2=88 and OCT4=42. 2A intervening sequences in the cassette also included additional 24 CpG motifs.

### Topological study of the reprogramming vector

**Fig.3:**
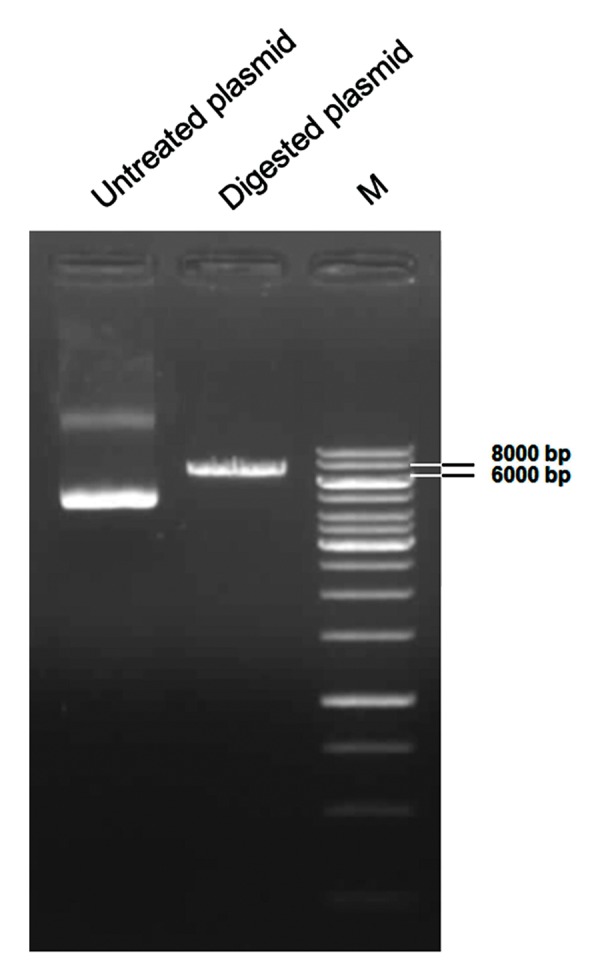
Analysis of isoforms of pLENSO/Zeo DNA and its stability by agarose gel electrophoresis. Electrophoresis was performed on a horizontal 1.0% agarose gel in TAE buffer at 60 V. The gel was then stained with ethidium bromide. The gel contained undigested plasmid, NotI-linearized plasmid and size marker, respectively. The intensities of the two resultant bands in the lane included undigested plasmid were quantified by ImageJ software. M; Molecular size marker (1 kb) Plus DNA Ladder (Thermo Scientific, USA).

In order to identify prominent isoforms of the vector, we analyzed pLENSO/Zeo DNA samples using agarose gel electrophoresis (Fig .3). The undigested original pDNA, applied to the first lane, separated into two major bands. The second lane contained NotI-linearized pLENSO/Zeo with a length of 6500 bp. According to previous studies (43), we concluded that the faster, more intense band corresponded to the supercoiled isoform and the second slower band was considered to be the open circle form of pDNA. Based on the intensity of the resultant bands measured by ImageJ software for untreated pDNA (lower band=4567.25 and upper band=1352.19) and digested one (6101.44), we estimated that approximately 82% of the pLENSO/Zeo plasmid was in the form of supercoiled DNA.

### Transfection and estimation of EGFP expression in HEK293 cells

**Fig.4:**
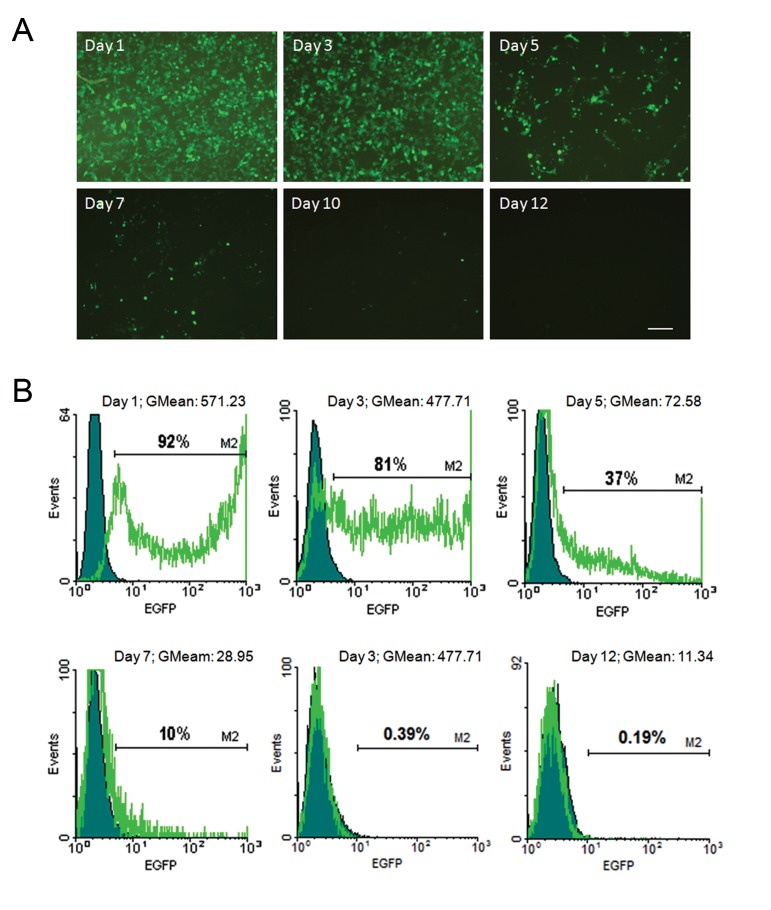
The EGFP-expressed by pLENSO/Zeo was monitored as a reporter signal in HEK293 cells during 12 days post-transfection using fluorescent microscope and flow cytometry. A. The figures show that the extrachromosomal vector was gradually lost in the pool of transfected cells that led to a reduction in the number of EGFP^+^ cells on days 1, 3, 5, 7, 10 and 12 post-transfection. Bar is 200 μm and B. Regular plasmid dilution in transfected cells was detected by flow cytometry on the mentioned days after transfection. Cells were analyzed based on green fluorescent signal. We also detected a time-mediated decrease in geometrical mean fluorescence intensity (MFI) from 571.23 to 11.34 during 12 days as presented in the histograms.

Due to its excellent transfectability, the HEK293 cell line is commonly used as an *in vitro* model for transfection and expression analysis of human transgene(s) (42). Therefore, we validated the functionality of our reprogramming cassette by transfecting the endotoxin- free vector into HEK293 cells. One day after transfection, we started monitoring the cells by fluorescent microscopy until the day 12 post-transfection (Fig .4A). According to the results, the transient transfection efficiency was estimated to be more than 90% after 24 hours. However, as expected, the GFP signal reduced over time via a gradual dilution and degradation of the vector due to several cell divisions, such that at day 3 post-transfection the frequency of EGFP^+^ cells reached to about 80%, but after 5 days decreased to 37% and finally completely disappeared after 12 days of transfection (Fig .4B).

### Analysis of genomic integration of the reprogramming vector

**Fig.5:**
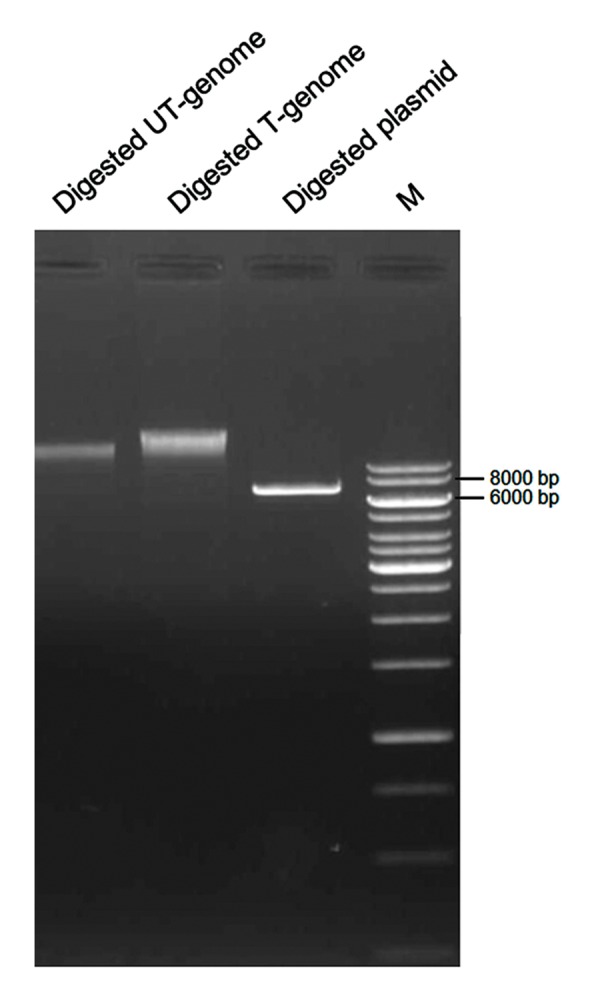
Isolation of extrachromosomal plasmid from the transfected genomic DNA. For this purpose, genomic DNAs of transfected and untransfected HEK293 cells were digested with NotI for 3 hours. As shown, if an amount of extrachromosomal pLENSO/ Zeo remained in the transfected cells, it could be isolated from digested genomic samples by gel electrophoresis because of the lower size of linearized pDNA (6.5 kb). T-genome; Transfected HEK cell-derived genome, UT-genome; Untransfected HEK cellderived genome, and M; Molecular size marker (1 kb) Plus DNA Ladder (Thermo Scientific, USA).

**Fig.6:**
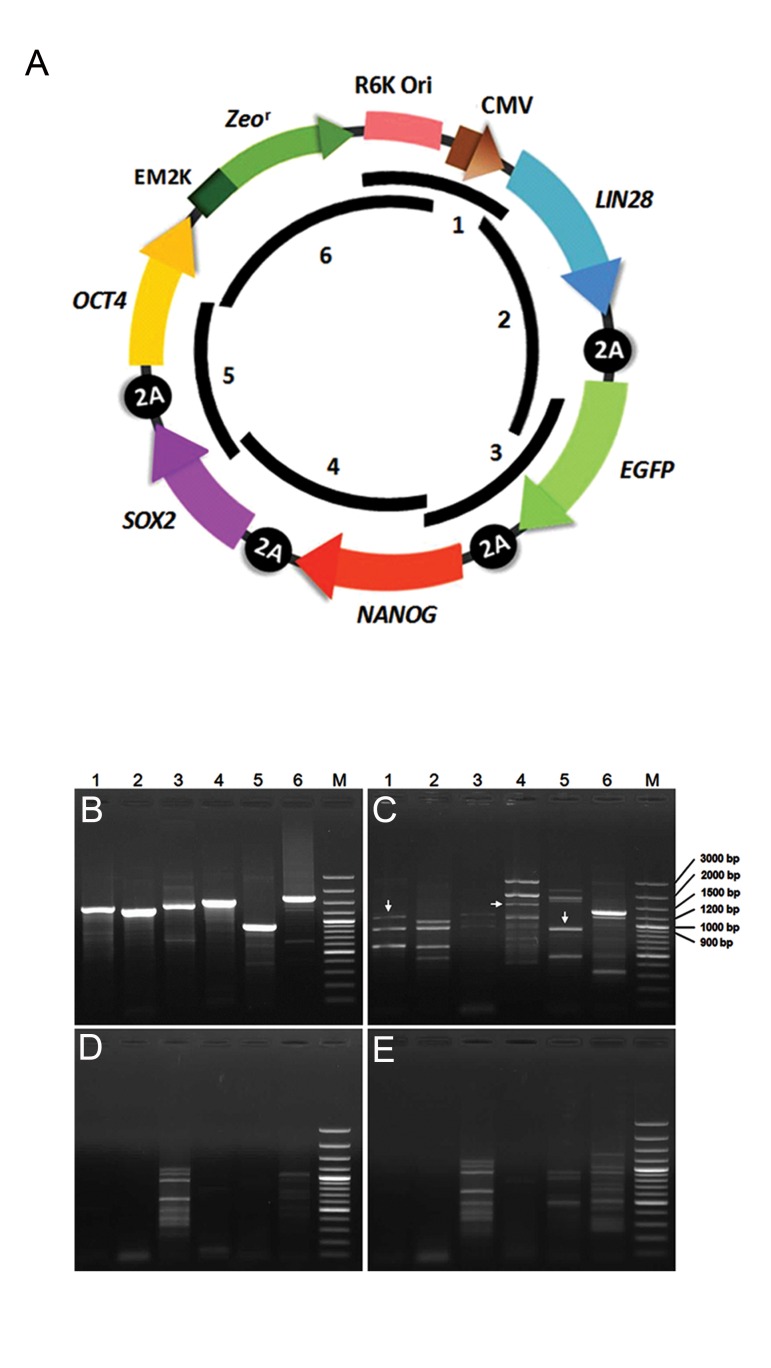
Determination of vector integration into the genome of transfected cells. In order to investigate the likely genomic integration of the vector, untransfected and transfected genomes at 12 days after transfection were digested with NotI. Genomic samples and plasmid DNA (as the positive control) were used for the PCR experiments. The list of primer pairs used for genomic polymerase chain reaction (PCR) analysis presented in Table 2. A. Schematic model of circular pLENSO/Zeo map showing the locations amplified by genomic PCRs. Thick lines inside the map with corresponding numbers indicate the relative positions of different parts of the vector amplified by six primer pairs listed in Table 2. The primers were used to detect likely integration of the pLENSO/Zeo into the cellular genome. Each primer pair overlaps with the previous and next ones to cover the whole vector sequence by genomic PCRs, B. PCR on the pLENSO/Zeo plasmid using six primer pairs showed amplification of different parts of the vector with the expected sizes, C. PCR was carried out on undigested genomic DNA of transfected cells. Some resultant faint bands suspected to have been derived from a number of vector segments according to their sizes (white arrowheads), D. PCR on digested genomic DNA of transfected cells after gel extraction. To isolate the traces of the vector that likely remain outside the chromosomes in transfected cells, we digested the genome with NotI and gel extracted. This genomic DNA sample was subsequently used for the PCR experiment, and E. NotI-digested genomic DNA from untransfected cells was also used as the negative control. The numbers above each lane indicate the primer pairs used in each experiment. M; Molecular size marker (100 bp) Plus DNA Ladder (Thermo Scientific, USA).

pLENSO/Zeo plasmid contains a unique restriction site for NotI. The enzyme also has minimal restriction site distributed throughout the human genome according to the NEB tool (http://tools.neb.com/~posfai/TheoFrag/TheoreticalDigest. human.html). Therefore, precise gel extraction of the NotI-digested genome of transfected cells could lead to exclusion of the unintegrated plasmid whose size was clearly smaller than genomic DNA (Fig .5). For this, transfected and untransfected genomes were digested with NotI to hinder amplification of trace amounts of pLENSO/Zeo that likely remained extrachromosomally. We designed six pairs of overlapping primers to amplify all sequences of the plasmid (Fig .6A). We performed the first PCR test on pLENSO/Zeo as the template using all primer pairs that amplified different fragments in the vector structure (Fig .6B). Also a sample of undigested transfected genome was employed for the second PCR. The results showed three suspect bands using primer pairs 1, 4 and 5, which were of similar size to those observed in the first PCR (Fig .6C). Then, gel-purified genomic DNAs were used as the template for additional rounds of PCR. The results showed that the suspected bands disappeared in PCR products of transfected genome and a similar pattern to the PCR product of untransfected genome (negative control) was obtained (Fig .6D, E). Although we cannot properly reject the presence of small fragments of the transfected plasmid, our results confirmed that the plasmid most likely did not integrate into the chromosomes and remained as an episome.

### Evaluation of reprogramming factors expression following transient transfection

**Fig.7:**
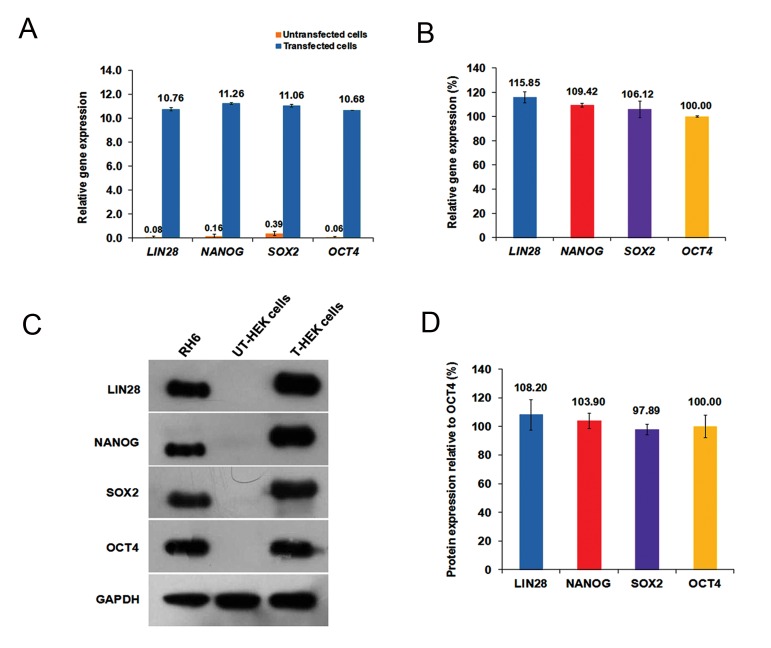
Evaluation of transgenic RFs expression kinetics in HEK293 cells at the RNA and protein levels. A. Quantification of the transcriptional activity of transgenic *LIN28, NANOG, SOX2* and *OCT4* in the pool of HEK293 cells transfected with pLENSO/Zeo. The graph shows RFs expression levels and related endogenous ones two days after transfection. Untransfected HEK293 cells were employed as the negative control for quantification of the endogenous transcripts. GAPDH was used as an internal control gene for normalization of the transgene expression levels, B. In this graph, the expression rate of OCT4 was considered 100%. The other transgenes were compared to OCT4. The data showed that expression levels of the transgenes in the reprogramming cassette were not significantly different at P<0.05, C. The representative Western blot analysis of polycistronic protein production showed active expressions of pLENSO/Zeo-based RFs proteins by transfected HEK293 cells compared to the untransfected ones (negative control) in comparable amounts to the ES cell line (Royan H6), and D. Estimation of transgenic RFs protein expression levels in HEK293 cells using Western blot analysis. For this, final images of Western blot membranes were acquired with a scientific grade CCD camera and used for band analysis. Quantification of the acquired bands and statistical analysis of their intensity exhibited no significant difference in protein expression level among RFs at P<0.05. Error bars signify standard error of mean (SEM) for three independent repeats. T; Transfected and UT; Untransfected.

We analyzed expression kinetics of the vectorencoded factors in transfected HEK293 cells at both RNA and protein levels by means of RT-qPCR and Western blot. To do this, we quantified the relative transcription rates of human *LIN28, NANOG, SOX2* and *OCT4* genes in the pool of transfected HEK293 cells to ensure equimolar expression. Analysis of RT-qPCR data offered clear evidence that not only all 2A-linked RFs were transcribed efficiently compared to untransfected cells, but their expression levels were not significantly different in transfected cells (Fig .7A, B). Consistently, Western blot analysis showed active expression of vector-based proteins for all RFs (Fig .7C). A shift in protein size of recombinant LIN28, NANOG and SOX2 was attributed to 2A-derived amino acids that remained attached at their carboxyl ends which resulted in slightly heavier transgenic proteins compared to those extracted from hESCs. Additionally, we did not observe any heavy protein band in sodium dodecyl sulfate polyacrylamide gel electrophoresis (SDS-PAGE). This indicated that no defect existed in 2A processing and verified the efficiency of the 2A peptide cleavage activity. Quantification of the Western blot data (Table 4) showed no significant differences in peptide expression levels among four RFs and confirmed the stoichiometric expression of RFs by the stem cassette (Fig .7D).

**Table 4:** Estimation of relative expression of reprogramming factors (RFs) peptide compared to OCT4


RF	LIN28			NANOG			SOX2			OCT4		

Sample no.	1	2	3	1	2	3	1	2	3	1	2	3
Optical intensity	722619	512513	649118	658943	598540	551784	610838	557205	536623	634806	671431	547543
RF/GAPDH ratio	1.25	0.89	1.13	1.14	1.04	0.96	1.06	0.97	0.93	0.91	1.17	0.95
Relative expression^a^	124.49	88.29	111.82	113.52	103.11	95.06	105.23	95.99	92.44	90.01	115.67	94.33
Mean^b^	108.20 ± 10.60			103.90 ± 5.34			97.89 ± 3.81			100.00 ± 7.93		


^a^; In each experiment, expressions of RFs were estimated relative to the OCT4 and ^b^; Data presented as mean ± SEM for three independent experiments.

## Discussion

We presented a rational design for construction and evaluation of a novel small, non-integrative plasmid based-vector, pLENSO/Zeo. This plasmid has a vector backbone free of CpG dinucleotides and a single reprogramming cassette that enabled accurate co-expressions of human *LIN28, NANOG, SOX2* and *OCT4* along with EGFP. In respect to RFs, we selected Thomson factors to avoid the use of c-MYC and KLF4 oncogenes. Expression of these factors potentially leads to DNA replication stress and genomic instability, which could presumably result in a cancerous state in the target cells. In addition, the role of c-MYC in enhancing the generation of partially reprogrammed cells has been previously reported (44). Absence of OCT4 and SOX2, as core RFs, hinders iPSC establishment, whereas NANOG and LIN28 are enhancers that increase the efficiency of reprogramming (45, 46). Previous study have demonstrated that differentiation efficiency of iPSCs and their subsequent applications signiﬁcantly rely on the number of transcription factors used for reprogramming (47). Regarding the order of the RFs in the structure of the polycistronic construct, we observed no significant differences at the mRNA and protein expression levels amongst the RFs. However, as OCT4 is the core RF and displays a crucial role in the reprogramming process, we have placed its ORF at the end of the cassette. By doing so, we restrained the addition of the 2A amino acids to the carboxyl end of the OCT4 protein that might influence its functionality. In order to achieve an effective reprogramming process by using a non-integrative vector, the reprogramming cassette should express RFs at high-levels over a limited time period. Consequently, our reprogramming cassette consisted of a human CMV immediate early enhancer-promoter, which derived a high constitutive expression level. Previous study reported the high cleavage efficiency of T2A sequences (48). This cleavage ability was also confirmed in our study, as only four corresponding bands of RFs were observed in the Western blot experiments. Since the rate of transcription is generally increased by the use of a strong promoter, the type of polyadenylation signal sequence in a vector significantly affects the transcription process (49). The combination of SV40PA signal with the CMV promoter has been demonstrated to provide a high-level transcription and improve the half-life of supercoiled pDNA in cell lysates (30). Hence, we added a SV40PA signal sequence at the end of the stem cassette. The orientations and compositions of prokaryotic elements could negatively affect the expression of a eukaryotic transgene (50). Williams et al. (51) have reported dramatically higher eukaryotic expression when the prokaryotic promoter was located distal from the CMV promoter. This feature was considered in our construct as the EM2K bacterial promoter was located distal to CMV.

In addition, small vectors are more effectively transfected into the target cells. An important advantage of reprogramming vectors may be their size, as a smaller plasmid carries more stem cassettes per unit weight of pDNA during transfection. This feature enhances the expression levels of RFs. Consequently, a shorter induction time and a higher reprogramming efficiency will be expected for smaller vectors. The pLENSO/Zeo contains four RFs and EGFP coding sequences that are 6.5 kb in size which can provide this quality.

According to Lu et al. (52), application of bacterial sequences of nearly 1000 bp or more in the structure of the vector causes transgene silencing. On the other hand, Hasse et al. (53) have constructed a vector which contained a relatively CpG-rich transgene unit within a CpG-depleted vector backbone that exhibited a similar expression level and duration when compared to minicircles. Therefore, to provide an accurate expression profile and avoid the risk of methylation-induced transgene silencing in our reprogramming cassette, we used a short CpG-free BB of 700 bp size from the pCpGfree-basic plasmid in the structure of pLENSO/Zeo. Conversely, Ball and colleagues have shown that gene-body methylation is associated with an enhanced expression level in highly expressed genes (54). Recent studies reveal a direct correlation of intragenic CpG content with transgene activity. Depletion of CpGs from the coding region of a transgene results in a significant reduction of *in vitro* expression, which suggests a methylation-independent role of intragenic CpGs in increasing expression level (55, 56). Based on these observations, we did not change the nucleotide sequence of the expression cassette because codon optimization resulted in decreased intragenic CpG content of the reprogramming cassette. Using wild type RFs, 230 intragenic CpG motifs were identified. As previously mentioned, the supercoiled pDNA is considered as biologically active conformation and the optimal isoform for transfection of mammalian cells. Thus, determination of the isoform distribution of pDNA is of great interest. The AT-rich domains were only restricted in two regions of the vector - R6Kγ ori and the SV40PA signal that confirmed the vector’s structural stability. Additionally, results of this study showed that the majority of the vector DNA (82%) was supercoiled. A transfection efficiency of more than 90% and average fluorescence intensity of more than 570 at one-day post-transfection in HEK293 cells has verified optimal DNA confirmation of the pLENSO/Zeo plasmid. This vector does not contain mammalian origin of replication; hence it cannot replicate in mammalian cells and is lost from the cells over cell division (57). Our experimental data confirmed this reality after 10-12 days of transfection. The chance of integration for these kinds of vectors is very low or unlikely, unless they become integrated into chromosomes. However, chromosomal integration by these non-replicating vectors is a very rare event, occurring with a probability of 1/103 to 1/105 and minimizing the risk of genomic integration (58, 59). PCR analyses of genomic DNA from the pool of transfected HEK293 cells has demonstrated that our vector remained in an extrachromosomal state in transfected cells. By monitoring the GFP signal, we showed that approximately 80% of the HEK293 cells expressed transgenes after 3 days of transfection. Accordingly, it seems that transfection process should be repeated every 3 to 4 days in order to preserve continuous expression of RFs in the target cells during the reprogramming process. In support of the functionality of our vector, expression of RFs by pLENSO/Zeo was measured in transiently transfected cells. According to the results of RT-qPCR, the vector-derived mRNA contained all transgene coding sequences and the transcription rates for *LIN28, NANOG, SOX2,* and *OCT4* in HEK293 cells were similar. Western blot analysis also showed no significant difference among the four vector-expressed discrete RFs proteins. Interestingly, the amounts of RFs were comparable to those produced in human ES cells as the positive control. Taken together, these findings confirmed the expression of recombinant RFs with almost equal amounts by pLENSO/Zeo.

## Conclusion

Here we introduced a new small, non-integrating nonviral vector named pLENSO/Zeo. It consisted of a CpG-depleted bacterial backbone to diminish tendency towards epigenetic silencing of the expression unit along with a CpG-rich multicistronic cassette of 254 CpG dinucleotides. The vector has shown the capability for stoichiometric, simultaneous, high-level production of RFs and the potential for use with other approaches such as various small chemical molecules and/or short interfering RNAs in order to improve the efficiency of iPSC generation. Therefore, pLENSO/Zeo can be considered as a simple, low cost tool for application in low-risk reprogramming and developing patient-specific or disease-specific cell lines for potential application in regenerative medicine or human disease modeling studies. However, the potential ability needs to be verified in future studies.
